# Surgery

**Published:** 1879-04

**Authors:** 


### SURGERY.

COTTON IMPREGNATED WITH CAMPHOR AND GLYCERINE FOR DRESSING WOUNDS.—M. Paoli. (*Journ. de Med. et Chi. prat. L'Union Médicale du Canada*, Jan., 1879.)

The author believes in the noxious influence of the air upon wounds, not only from the germs which it contains, but from the dust, and even the oxygen and nitrogen, and in general whatever it may contain. To protect wounds from its influence he recommends the following: wash the wounds with aqueous solution of carbolic acid, 1 part to five hundred; cover with a thick and large layer of cotton first moistened aud then impregnated with glycerine, the wound being first bathed with glycerine, and then sprinkled with powdered camphor. Over the wet cotton a thick layer of dry cotton is placed, and the dressing is rarely renewed, except the first few days when it may be re-applied every three or four days. Under this dressing the aspect of the wounds is excellent, and the cicatrization goes on regularly and without accident.

As examples of the value of this treatment the author cites several interesting cases ; a case of gun-shot wound of the scrotum, a penetrating wound of the skull, a fracture of the sacrum by gun-shot, the ball entering the pelvis, a compound fracture of the leg, a penetrating wound of the knee, amputation of the leg, etc., etc. In anfractuous wounds he injects glycerine.

*A priori*, this dressing which the author also applies to simple contusions should produce the good results proved by experience. Its three elements, camphor, glycerine and cotton, are all excellent protectors for wounds. Camphor is an excellent antiseptic. Glycerine is also of much value as a topical remedy. Guyon uses with all the precautions of the antiseptic method, cotton saturated with carbolized glycerine, and has excellent results.

DENTAL GRAFTING.—(*Archives Générales de Médecine*, Jan., 1879.)

We have already called attention to the inaugural thesis of Dr. David on dental grafting. (*Arch.*, Feb., 1878.) In support of the method exposed in his remarkable work, the author now communicates to the Academy a series of observations which testify to its value. Twenty times he has applied it to teeth recognized as incurable ; he has had but one failure. To extract a tooth, subject it to an operation impracticable in the mouth, reinstate it in its alveola ; such is the method the operator follows. In ten to twelve days, on an average, the tooth is grafted by the intermediary of the alveolo-dental periosteum, has regained its solidity, and is cured as well as the secondary affections of which it was the point of departure. This has been proved in an evident manner by one of the most interesting cases in which, with the view of reducing an anomaly of direction, a tooth had to be extracted and replaced twice ; this case furnished the rare occasion of proving the renewal of the vascular connections of the pulp. Here then is an operation destined, thanks to these later labors, to transform dental therapeutics.

